# Elucidating the Molecular Mechanism of Ischemic Stroke Using Integrated Analysis of miRNA, mRNA, and lncRNA Expression Profiles

**DOI:** 10.3389/fnint.2021.638114

**Published:** 2021-08-16

**Authors:** Yaxuan Sun, Jing Wang, Bin Han, Kun Meng, Yan Han, Yongxia Ding

**Affiliations:** ^1^Department of Neurology, Shanxi People’s Hospital, Taiyuan, China; ^2^College of Nursing, Shanxi Medical University, Taiyuan, China

**Keywords:** ischemic stroke, miRNA, lncRNA, mRNA, regulatory axis

## Abstract

**Objective:** This study aimed to investigate the possible molecular mechanisms associated with ischemic stroke through the construction of a lncRNA-miRNA-mRNA network. miRNA expression profile in GSE55937, mRNA and lncRNA expression profiles in GSE122709, and mRNA expression profile in GSE146882 were downloaded from the NCBI GEO database. After the identification of the differentially expressed miRNA, lncRNA, and mRNA using GSE55937 and GSE122709 in ischemic stroke *vs.* control groups, a protein-protein interaction (PPI) network was constructed. The lncRNA-miRNA, lncRNA-mRNA, and miRNA-mRNA pairs were predicted, and a lncRNA-miRNA-mRNA network was constructed. Additionally, the gene-drug interactions were predicted. Characteristic genes were used to construct a support vector machine (SVM) model and verified using quantitative reverse transcription polymerase chain reaction. In total 38 miRNAs, 115 lncRNAs, and 990 mRNAs were identified between ischemic stroke and control groups. A PPI network with 371 nodes and 2306 interaction relationships was constructed. The constructed lncRNA-miRNA-mRNA network contained 7 mRNAs, 14 lncRNAs, such as SND1-IT1, NAPA-AS1, LINC01001, LUCAT1, and ASAP1-IT2, and 8 miRNAs, such as miR-93-3p and miR-24-3p. The drug action analysis of the seven differential mRNAs included in the lncRNA-miRNA-mRNA network showed that four genes (*GPR17*, *ADORA1*, *OPRM1* and *LPAR3*) were predicted as molecular targets of drugs. The area under the curve of the constructed SVM model was 0.886. The verification results of the relative expression of RNA by qRT-PCR were consistent with the results of bioinformatics analysis. *LPAR3*, *ADORA1*, *GPR17*, and *OPRM1* may serve as therapeutic targets of ischemic stroke. lncRNA-miRNA-mRNA regulatory axis such as SND1-IT1/NAPA-AS1/LINC01001-miR-24-3p-*LPAR3*/*ADORA1* and LUCAT1/ASAP1-IT2-miR-93-3p*-GPR17* may play important roles in the progression of ischemic stroke.

## Introduction

Ischemic stroke, characterized by cerebral ischemia, represents a leading cause of long-term disability and mortality around the world (Barthels and Das, 2020), and its incidence has been increasing. Globally, about 15 million people suffer from stroke each year and ischemic stroke accounts for around 87% of these cases (Bao et al., 2018). Ischemic stroke is a highly complex disease with multiple risk factors, such as atherosclerosis, hypertension, alcohol consumption, smoking, and type 2 diabetes (Tmoyan et al., 2020). Currently, thrombolysis is the only effective treatment for ischemic stroke (Campbell, 2017). Despite the advances in the understanding of the cause and treatment of this disease, its exact pathophysiologic mechanism is still not completely understood. Thus, a better understanding of the molecular mechanisms underlying the pathological process of stroke may provide new methods for ischemic stroke therapy.

Recently, it was reported that genetic factors are associated with stroke risk and some genetic variations have been discovered through genome-wide association studies (Chen et al., 2019). Claudin-5, as a tight link protein in the blood-brain barrier (BBB), is involved in regulating the integrity and permeability of BBB. Increasing the expression of claudin-5 can play a protective role in neurological diseases, especially in ischemic stroke (Lv et al., 2018). The brain after ischemia is mainly characterized by severe inflammation. Interleukin 1 and tumor necrosis factor are elevated in peripheral blood leukocytes within a few hours after ischemic stroke (Rostevanov et al., 2020). In addition to coding RNAs, some non-coding RNAs, including microRNAs (miRNAs) and long non-coding RNAs (lncRNAs), also play critical roles in the pathology of ischemic stroke. For instance, some miRNAs, such as miR-155, miR-125a/b-5p, and miR-22, can regulate blood pressure and therefore affect patients’ outcomes during ischemic stroke therapy (Bulygin et al., 2020). Hyperglycemia in patients with type 2 diabetes induces platelet activation through miR-144 and miR-233. Low expression levels of platelet and plasma miR-233 and high expression levels of platelet and plasma miR-144 may be risk factors for ischemic stroke in patients with type 2 diabetes (Yang et al., 2016). The roles of lncRNAs in ischemic stroke have recently been elucidated, and many aberrantly expressed lncRNAs have been identified, such as MALAT1 (Zhang et al., 2017), MEG3 (Yan et al., 2016), and H19 (Wang et al., 2017). It is necessary to understand the effects of lncRNA-miRNA-mRNA axis on ischemic stroke.

In this study, we conducted an integrated analysis of the miRNA, mRNA, and lncRNA expression profiles of ischemic stroke. After the identification of the differentially expressed miRNAs, mRNAs, and lncRNAs in ischemic stroke group *vs.* healthy group, the lncRNA-miRNA, lncRNA-mRNA, and miRNA-mRNA pairs were predicted, and a lncRNA-miRNA-mRNA network was constructed. The results may further explain the regulatory mechanisms of miRNAs and lncRNAs and provide new therapeutic strategies against ischemic stroke.

## Materials and Methods

### Expression Profile Data

Three expression profile datasets (GSE55937, GSE122709, and GSE146882) were downloaded from the GEO database.^1^ GSE55937 is a miRNA expression profile, involving 48 plasma samples (24 ischemic stroke patients *vs.* 24 healthy adults) and was detected on the platform of GPL16384 [miRNA-3] Affymetrix Multispecies miRNA-3 Array. GSE122709 is an expression profile of mRNAs and lncRNAs, involving 5 ischemic stroke patients and 5 healthy adults, and the detection platform was GPL20795 HiSeq X Ten (Homo sapiens). GSE146882 is an expression profile of mRNAs, involving ten patients with atherosclerosis-induced ischemic stroke and ten healthy volunteers, and the detection platform was GPL23178 [OElncRNAs520855F] Affymetrix Human Custom lncRNA Array. GSE55937 and GSE122709 were selected to screen differentially expressed mRNAs, lncRNAs, and miRNAs, while GSE122709 and GSE146882 were selected to construct the support vector machine (SVM) model.

### Data Preprocessing

The expression matrixes of miRNA microarray at 24 h were analyzed. The miRNA was reannotated using the platform annotation file. For a miRNA corresponding to multiple probes (expression values), the mean expression value of the miRNA was considered. For the profile data of GSE122709, the human reference genome [Release 32 (GRCh38.p13)] annotations file (gencode.v32.annotation.gtf) was downloaded from the GENCODE database (Frankish et al., 2018).^2^ The symbol with the annotation information of “protein_coding” was reserved as mRNA, while the symbol with the annotation information of “lncRNA” was reserved as lncRNA. For the mRNA data of GSE146882, preprocessed and normalized matrix of probe expression values and annotation information were downloaded. Probes with no match to gene symbols were eliminated. For different probes mapped to the same gene, we considered the mean of the different probes as the final expression value for this gene.

### Identification of Differentially Expressed mRNAs, lncRNAs, and miRNAs

Using the linear regression and empirical Bayes method provided by R (v3.6.1) and limma (v3.42.0) (Ritchie et al., 2015)^3^ packages, the expression matrixes of miRNA microarray from the Affymetrix platform were subjected to differential expression analysis. The *p* values of corresponding expression differences were determined. The differential expression thresholds of miRNA were set as *p* value <0.05 and | log fold change (FC)| > 0.263. The result has not been corrected. The trimmed mean of M-values algorithm (Robinson and Oshlack, 2010) in R package edgeR (v3.28.0) (Robinson et al., 2010) was used to calculate the normalized factors, ordinary dispersion, and intra-gene dispersion of mRNA and lncRNA expression profiles on the Hiseq platform. Additionally, the exact test was performed to obtain the differential expression results and *p* value. The expression thresholds of lncRNA and mRNA were false discovery rate (FDR) < 0.05 and | logFC| > 2.

### PPI Network and Module Analyses of Differentially Expressed mRNAs

The interaction between differentially expressed mRNA was predicted using String database (v11) (Szklarczyk et al., 2018),^4^ and the relationship with confidence score > 0.9 (highest confidence) was determined. The network was built using Cytoscape v3.7.2 (Shannon et al., 2003).^5^ The degree values of nodes were counted and the connections between nodes were observed. The functional modules were predicted using plug-in MCODE (v1.5.1) (Bader and Hogue, 2003),^6^ and the module with score >10 was selected for gene ontology-biological processes (GO-BP) enrichment analysis.

### Co-expression Analysis of lncRNAs and mRNAs

Based on the expression data and Pearson correlation coefficient method, the co-expression relationship between differentially expressed lncRNAs and mRNAs was determined, and the correlation test was conducted to screen the lncRNA-mRNA relationship with a co-expression threshold of | r| > 0.95 and a *p* value <0.05. According to all the selected positive co-expression relationships, the lncRNAs that had co-expression relationships with differentially expressed mRNAs in the functional modules were selected for GO-BP enrichment analysis using clusterProfiler R package (v3.14.0) (Yu et al., 2012).^7^

### Prediction of Target Genes for Differentially Expressed miRNAs

The miRNA target gene prediction tool miRWalk v3.0 (Sticht et al., 2018) was used to predict the differentially expressed mRNAs that were related to differentially expressed miRNAs and existed in the functional module predicted by MCODE. The miRNA regulatory network was established, and the target genes were all differentially expressed mRNAs. Additionally, the miRNA-mRNA regulatory relationships must also be validated in at least one of the following databases: mirdb release 6.0 (Wong and Wang, 2014), mirtarbase release 7.0 (Chou et al., 2017), and TargetScan release 7.2 (Agarwal et al., 2015). To study the BP regulated by these miRNAs, the differentially expressed miRNAs that regulated the mRNAs in functional modules were extracted. Besides, the differentially expressed mRNAs in the PPI network that were regulated by the aforementioned differential miRNAs were also selected. According to the mRNA regulated by each miRNA, BP terms regulated by miRNA were predicted through GO-BP enrichment analysis.

### Prediction of lncRNA-miRNA Relationship and lncRNA-miRNA-mRNA Network Analysis

DIANA-LncBase (v2) database (Agarwal et al., 2015) was used to identify differentially expressed lncRNAs related to miRNAs that regulated the module genes. The lncRNA-miRNA regulatory relationships with thresholds greater than 0.7 were screened.

The obtained lncRNA-miRNA, lncRNA-mRNA (positive co-expression), and miRNA-mRNA interactions were integrated to construct the lncRNA-miRNA-mRNA regulatory network of lncRNA, miRNA, and mRNA using Cytoscape.

### Drug and Transcription Factor (TF) Prediction

For mRNAs in the lncRNA-miRNA-mRNA network, drug prediction was performed using DGIgb (v3.0) (Cotto et al., 2017).^8^ Moreover, the TFs that regulated the expression levels of differentially expressed mRNAs were predicted through TRUSST v2 (Han et al., 2017).^9^

### SVM Model Prediction

In order to determine whether the gene can be used as biomarker in clinic. GSE122709 and GSE146882 including 15 ischemic stroke patients and 15 healthy participants were selected to construct the SVM model. The expression values of four characteristic genes (*LPAR3*, *ADORA1*, *GPR17, OPRM1*) in two groups of samples were used as the characteristic values for classification prediction using SVM classifier of R package e1071 (v1.7-3) (Paraskevopoulou et al., 2015). The classification and prediction efficiencies of the model were evaluated through receiver operating characteristic (ROC) curve.

### RNA Extraction and Quantitative Real-Time Polymerase Chain Reaction (qRT-PCR)

The extraction of total RNA from the peripheral blood of 10 ischemic stroke patients (ischemic stroke group) and 10 volunteers (normal control group) was carried out using the Trizol reagent (10296-010, Invitrogen, United States). PrimeScript Rt reagent Kit with gDNA Eraser (RR047A, Takara, Japan) was used to synthesize cDNA. We performed qPCR using TB Green^®^ Premix Ex Taq^TM^ II (RR820A, Takara, Japan), according to the specification provided by the manufacturer, on Applied Biosystems 7500 quantitative PCR instrument (Applied Biosystems, United States). The relative expression levels of RNAs were calculated using the 2^–ΔΔ*Ct*^ method. Two-tailed Student’s *t* test was used to significance of differences between two groups. Statistical significance was set at *p* < 0.05. *GAPDH* and RNU6B (U6) were set as internal control. The primer sequences are listed in Supplementary Table 1.

The studies involving human participants were reviewed and approved by Ethics Committee of Shanxi Provincial People’s Hospital (2021-17). The participants provided their informed consent.

## Results

### Differential Expression Analysis

**FIGURE 1:**
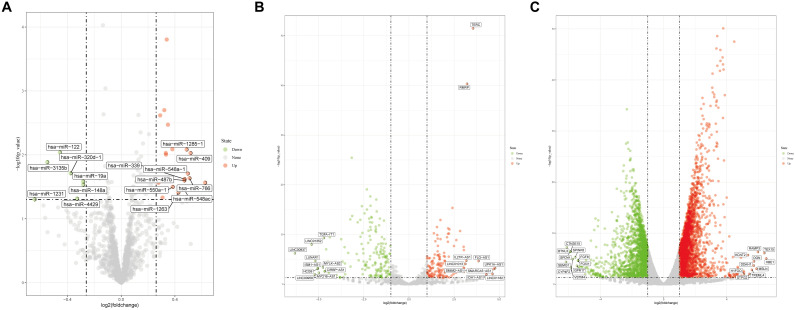
Volcano plots for **(A)** differentially expressed miRNAs, **(B)** differentially expressed lncRNAs, and **(C)** differentially expressed mRNAs.

After reannotation, a total of 1733 miRNAs, 2258 lncRNAs, and 18,669 mRNAs were obtained. Using | logFC| > 0.263 and *p* < 0.05 as the thresholds, 38 miRNAs (31 upregulated and 7 down-regulated miRNAs) were selected; at | logFC| > 2 and *p* < 0.05, 115 lncRNAs (37 upregulated and 78 downregulated) and 990 mRNAs (547 upregulated and 443 downregulated) were identified. Volcano plots of differentially expressed miRNAs, lncRNAs, and mRNAs are shown in Figure 1.

### PPI Network Analysis

**FIGURE 2:**
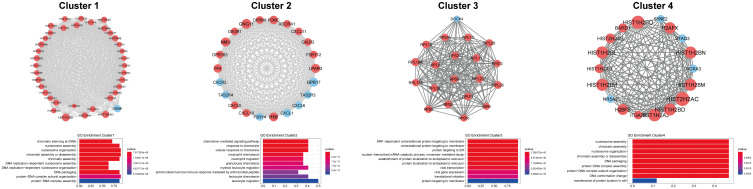
Four PPI networks and biological processes enrichment results. Size of node represents degrees. Nodes with blue are downregulated, and those with red are upregulated.

The PPI network of differentially expressed mRNAs obtained from String had a total of 371 nodes and 2306 interaction relationships. After the functional module analysis of MCODE, four clusters were suggested as functional modules (scores >10) (Figure 2). In cluster 1, there were 38 upregulated and one downregulated mRNAs; in cluster 2, there were 15 upregulated and seven downregulated mRNAs; in cluster 3, there were 19 upregulated and one downregulated mRNAs; and in cluster 4, there were 14 upregulated and four downregulated mRNAs.

Function analysis showed that genes in cluster 1 were significantly associated with chromatin silencing at rDNA; those in cluster 2 were significantly enriched in chemokine-mediated signaling pathway and response to chemokine; those in cluster 3 were significantly enriched in SRP-dependent cotranslational protein targeting to membrane and protein targeting to ER; and those in cluster 4 were significantly enriched in nucleosome assembly and chromatin assembly (Figure 2).

### Prediction of lncRNA-mRNA Co-expression Relationships

**FIGURE 3:**
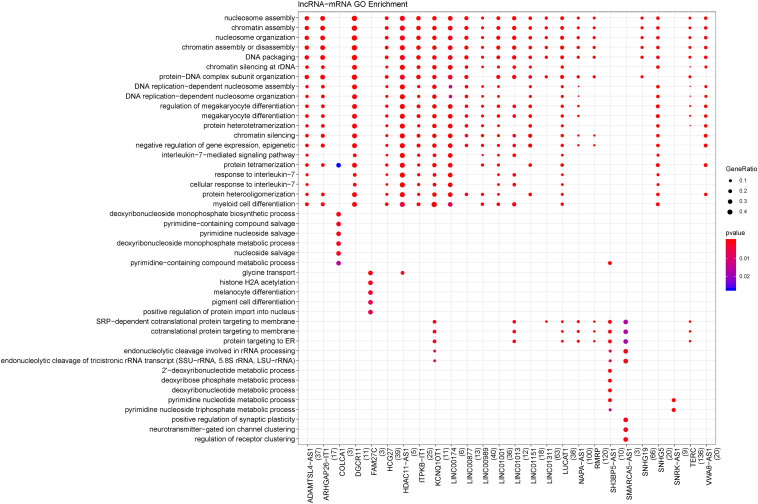
Biological processes enriched by lncRNAs. The changes in bubble color from blue to red indicate that the correlation is significant from low to high. Dot size GeneRatio is gene proportion. The size of the gene specific proportion indicates the proportion of the enrichment item.

There were 1947 differentially expressed lncRNA-mRNA pairs, of which 1387 were positively correlated, including 94 lncRNAs and 563 mRNAs. In addition, 36 lncRNAs had co-expression relationships with 79 mRNAs in the functional modules. Based on all the positively correlated co-expression relationships of lncRNA and mRNA, the functions of the 36 differentially expressed lncRNAs were predicted. As shown in Figure 3, the BP terms nucleosome assembly, chromatin assembly, DNA packaging, protein-DNA complex subunit organization, etc., were significantly enriched.

### miRNA-Target Gene Prediction

**FIGURE 4:**
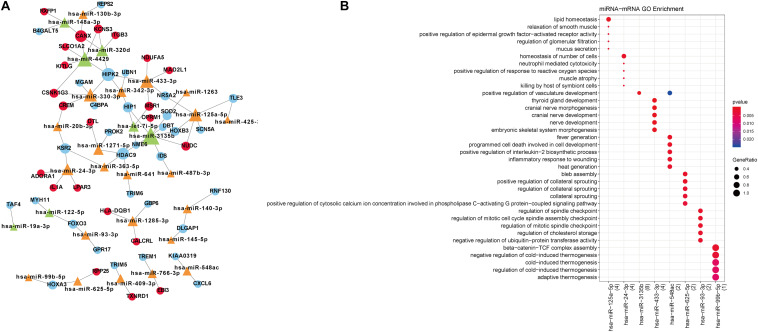
**(A)** Differentially expressed miRNA-target network. Blue circular nodes are downregulated mRNAs; Red circular nodes are upregulated mRNAs; Green triangles are downregulated miRNAs; Orange triangles represent upregulated miRNAs. **(B)** The biological processes enriched by miRNAs. The changes in bubble color from blue to red indicate that the correlation is significant from low to high. Dot size GeneRatio is gene proportion. The size of the gene specific proportion indicates the proportion of the enrichment item.

A total of 80 miRNA-target gene relationship pairs were identified by miRWalk v3.0, including 29 differentially expressed miRNAs and 53 differentially expressed mRNAs (Figure 4A). Among the 80 miRNA-target gene relationship pairs, nine pairs comprised seven differentially expressed mRNAs in functional modules. The nine miRNA-target gene pairs included eight miRNAs, which were significantly enriched in lipid homeostasis, cranial nerve morphogenesis, and programmed cell death involved in cell development (Figure 4B).

### lncRNA-miRNA-mRNA Network Analysis

**FIGURE 5:**
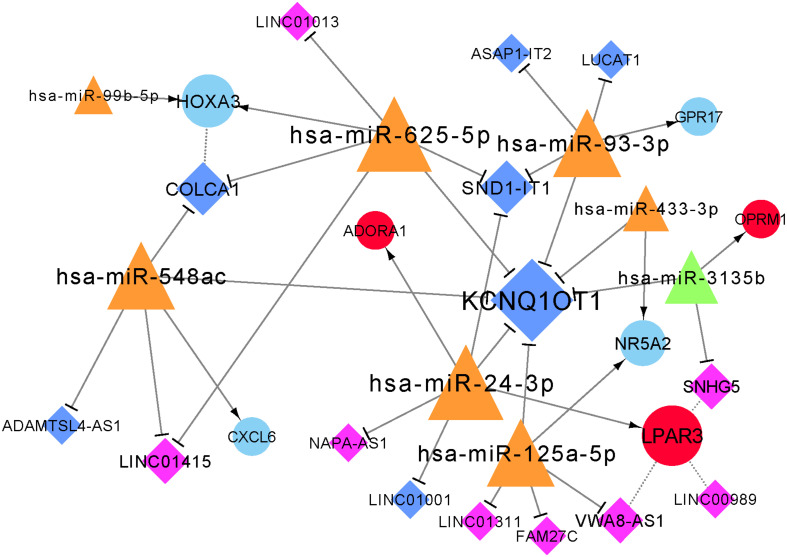
Constructed lncRNA-miRNA-mRNA network. Red circular nodes are upregulated mRNAs and blue circles are downregulated mRNAs. Dark-blue diamonds represent downregulated lncRNAs and purple diamonds represent upregulated lncRNAs. Orange triangles represent upregulated miRNAs and green triangles are downregulated miRNAs. Dotted lines demonstrate positively-correlated interactions of mRNAs and lncRNAs, straight lines with arrow represent interactions between miRNAs and mRNAs, and a straight line with T arrow means lncRNA-miRNA interaction. The size of node represents its degree.

The constructed lncRNA-miRNA-mRNA network contained seven mRNAs, 14 lncRNAs, and eight miRNAs (Figure 5). Additionally, the network included four positively correlated lncRNA-target (mRNA), 24 lncRNA-miRNA interactions, and nine miRNA-mRNA interactions.

### Drug and TF Prediction for mRNA in lncRNA-miRNA-mRNA Network

**FIGURE 6:**
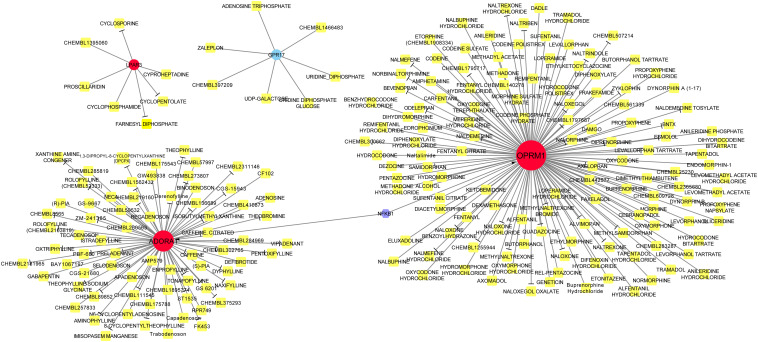
Drug-target-transcription factor (TF) network. Red circles represent upregulated genes, whereas blue circle represents downregulated genes. Yellow squares are drugs and the purple hexagon is the TF. T-arrow indicates the drug acts as an antagonist to this specific molecular target.

Drug action analysis was performed for seven differential mRNAs included in the lncRNA-miRNA-mRNA network, and four genes were predicted as molecular targets of drugs, involving 213 drug-target pairs (Figure 6). We found that lysophosphatidic acid receptor 3 (LPAR3) and G protein-coupled receptor 17 (GPR17) were targeted by seven drugs. Adenosine A1 receptor (ADORA1) was targeted by 71 drugs and opioid receptor Mu 1 (OPRM1) was targeted by 128 drugs. Among the drugs, 76 acted as antagonists, which may have inhibiting effect on the related biological processes of OPRM1, ADORA1, and LPAR3. TF prediction of the seven mRNAs identified only one TF [nuclear factor kappa B subunit 1 (NFKB1)], which regulated the transcription of OPRM1 and ADORA1.

### SVM Model Prediction

**FIGURE 7:**
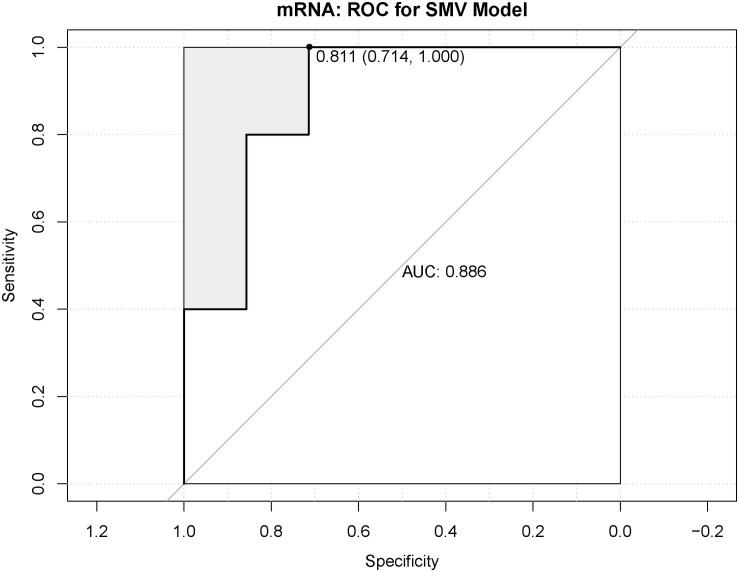
Receiver operating characteristic (ROC) analysis of support vector machine (SVM) model.

In order to determine whether the molecular targets of drugs can be used as biomarkers for clinical application. Four genes, LPAR3, ADORA1, GPR17, and OPRM1, were identified by SVM model prediction as molecular targets of drugs and ROC curve was used to predict the efficiency of the model. As shown in Figure 7, the area under the curve (AUC) was 0.886.

### qRT-PCR Verification

**FIGURE 8:**
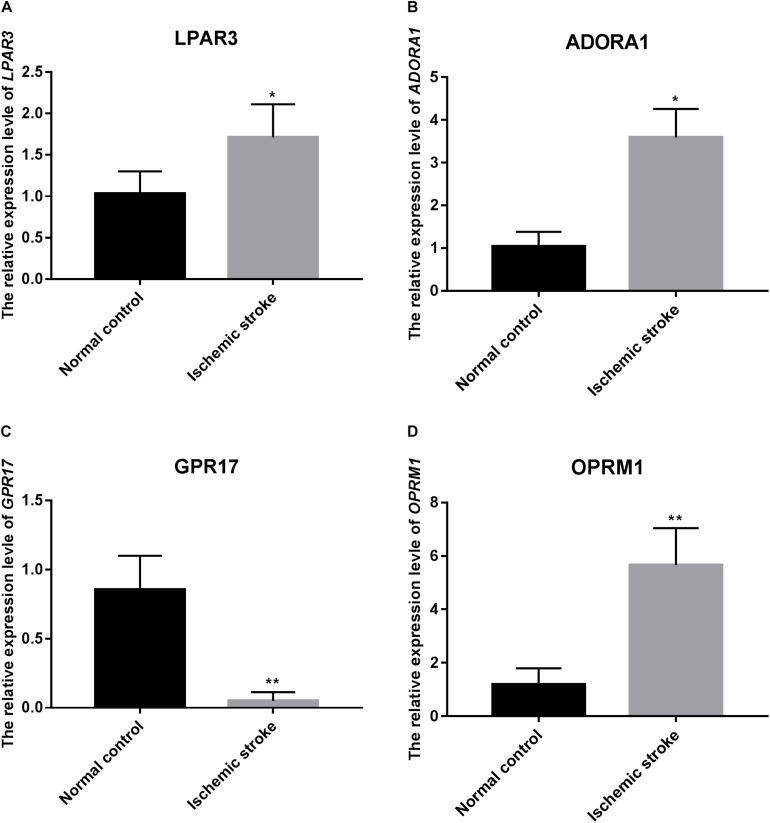
The relative expression level of *LPAR3* **(A)**, *ADORA1* **(B)**, *GPR17* **(C)**, and *OPRM1* **(D)**. * represents *p* < 0.05, and ** represents *p* < 0.01.

The relative expression level of four genes used for the construction of the SVM model was verified using qRT-PCR. As predicted, the relative expression level of GPR17 in the ischemic stroke group was significantly lower than that in the normal group (*p* < 0.01), whereas the relative expression of LPAR3 (*p* < 0.05), ADORA1 (*p* < 0.05), and OPRM1 (*p* < 0.01) in the ischemic stroke group was significantly higher than that in the normal group (Figure 8).

**FIGURE 9:**
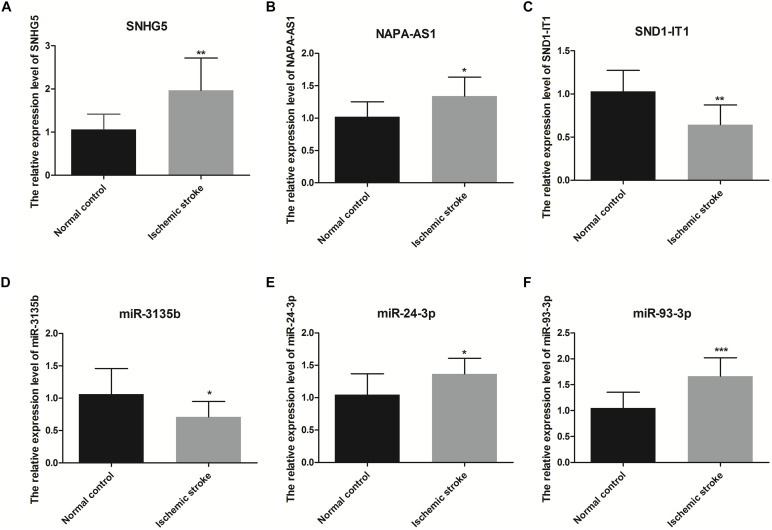
The relative expression level of SNHG5 **(A)**, NAPA-AS1 **(B)**, SND1-IT1 **(C)**, miR-3135b **(D)**, miR-24-3p **(E)**, and miR-93-3p **(F)**. * represents *p* < 0.05, and ** represents *p* < 0.01.

qRT-PCR was also used to verity the relative expression level of RNAs related with that four RNAs which were used to construct SVM model (Figure 9). These RNAs included three lncRNAs (SNHG5, NAPA-AS1, SND1-IT1) and three miRNAs (miR-3135B, miR-24-3p, miR-93-3p). As shown in Figure 9, the relative expression of SNHG5, NAPA-AS1, miR-24-3P, and miR-93-3p in the ischemic stroke group was significantly higher than that in the control group (*p* < 0.05), whereas the relative expression of SND1-IT1 and miR-3135b in the ischemic stroke group was significantly lower than that in the control group (*p* < 0.05).

## Discussion

In this study, based on the differentially expressed miRNAs, mRNAs, and lncRNAs obtained from the plasma of ischemic stroke patients, a lncRNA-miRNA-mRNA network was constructed. Among the seven mRNAs in the network, four genes (*LPAR3*, *ADORA1*, *GPR17*, and *OPRM1*) were predicted to have interactions with drugs. They were associated with the regulatory axis, such as SND1-IT1/NAPA-AS1/LINC01001-miR-24-3p-*LPAR3*/*ADORA1* and LUCAT1/ASAP1-IT2-miR-93-3p*-GPR17*, which may play important roles in the progression of ischemic stroke.

Both *LPAR3* and *ADORA1* were regulated by miR-24-3p in the network. Additionally, miR-24-3p interacted with four lncRNAs, including KCNQ1OT1, SND1-IT1, NAPA-AS1, and LINC01001. LPAR3 is a receptor for lysophosphatidic acid (LPA), which is a multi-functional glycerophospholipid. LPA affects six G protein-coupled receptors (Gaire et al., 2019). Among these receptors, *LPA1* and *LPA5* have been identified as the pathogenic factors of acute ischemic injury (Gaire et al., 2019; Sapkota et al., 2020). It has been reported that *LPA5* is upregulated in the injured brain after acute ischemic stroke, and inhibiting its activity can reduce acute brain injury by reducing the inflammatory response in the injured brain (Sapkota et al., 2020). In this study, the expression of *LPAR3* increased in patients with ischemic stroke, but there has been no prior report on the relationship between *LPAR3* and ischemic stroke. The progression of asymptomatic carotid artery stenosis (ACAS) in patients with luminal stenosis >50% is considered a potential risk factor for ischemic stroke. A study reported that the expression of miR-24-3p significantly increased in patients with ACAS progression (Dolz et al., 2017), which is consistent with our result that the expression of miR-24-3p was clearly upregulated in patients with ischemic stroke compared with healthy control. Intracranial atherosclerosis is a common cause of ischemic stroke and has a high recurrence rate (Hurford and Rothwell, 2021). In a prospective study, miR-24-3p was identified to be significantly associated with angiogenic factors, which are associated with intracranial atherosclerosis (Jiang et al., 2019).

The relative expression levels of SND1-IT1 in the ischemic stroke group was significantly lower than that in the control group, whereas the relative expression of NAPA-AS1 in the ischemic stroke group was significantly higher than that in the control group. The result of qRT-PCR verification is consistent with the result of our bioinformatic analysis. In this study, NAPA-AS1, KCNQ1OT1 and LINC01001 were identified as possibly involved in chromatin and nucleosome assembly. A previous study revealed that prothymosin α could stimulates cell proliferation and differentiation through chromatin remodeling and was shown to be protective against ischemic stress (Krishna et al., 2015). Extracellular nucleosomes were recently shown to promote coagulation and intravascular thrombus formation (Massberg et al., 2010). Elevated concentrations of DNA and nucleosomes have also been found in stroke patients (De Meyer et al., 2012). These results indicated that NAPA-AS1, KCNQ1OT1 and LINC01001 may also involved in the process of ischemic stress by regulating the chromatin and nucleosome assembly. More experiments are needed in the future. Thus, we speculated that KCNQ1OT1, SND1-IT1, NAPA-AS1, and LINC01001 may interact with miR-24-3p and facilitate the regulation of *LPAR3* and *ADORA1* in ischemic stroke.

*GPR17* was regulated by miR-93-3p, which interacted with four lncRNAs, such as LUCAT1 and ASAP1-IT2. GPR17 belongs to a G protein-coupled receptor superfamily, which is the largest and most diverse cell surface receptors (Marucci et al., 2019). It has been reported that *GPR17* mediates immune response and ischemic/inflammatory states, including stroke and some neurodegenerative diseases (Zhao et al., 2018) *GPR17* receptor may be a target for stroke, brain and spinal cord injury, and diseases characterized by neuronal and myelin dysfunctions (Bonfanti et al., 2017, 2020; He et al., 2018). We found that the relative expression level of *GPR17* in ischemic stroke patients was significantly lower than that in normal participants (*p* < 0.01). Bonfanti et al. reported that GPR17 is transiently expressed on early oligodendrocyte precursors, and has emerged as a target for implement stroke repair through the stimulation of oligodendrocyte precursors maturation (Bonfanti et al., 2017). The verification result of the expression level of miR-93-3p is consistent with the result predicted by bioinformatics. Thus, we speculated that miR-93-3p may be involved in the progression of ischemic stroke by regulating *GPR17*.

Additionally, *OPRM1* and *ADORA1* were predicted to be regulated by TF NFKB1. NFKB is a main transcription regulator of apoptosis, cell growth, and genes associated with immune response control, and it plays a key role in the modulation of inflammation (Zhang et al., 2016). Interestingly, evidence has indicated that inflammation plays a critical role in ischemic stroke (Shekhar et al., 2018). After an ischemic stroke, secondary neuroinflammation occurs, and specifically, pro-inflammatory signals from immune mediators quickly cause a large number of inflammatory cells to infiltrate the ischemic area, thereby aggravating brain damage (Jayaraj et al., 2019). *NFKB1* gene encodes a 105 kD non-DNA-binding protein of NFKB, which undergoes cotranslational processing to produce a 50-kD DNA-binding protein (p50) (Cartwright et al., 2016). The p50 subunit has both pro- and anti-inflammatory properties. A recent study has indicated that the polymorphisms in NFKB1 promoter can modulate the susceptibility to ischemic stroke (Kim et al., 2018). Moreover, *OPRM1* and *ADORA1* were involved in cluster 2, and this cluster was associated with some inflammatory functions (De Gregori et al., 2015; Borea et al., 2018). Taken together, *OPRM1* and *ADORA1* may be associated with the susceptibility of ischemic stroke through the inflammatory pathway.

There were some limitations in this study. On the one hand, we only used qRT-PCR to verify the expression level of RNA, and research on the mechanism requires more experiments in the future study. On the other hand, the data sets included in this study came from different testing platforms, which may cause our results to deviate from the results produced by using the same platform data.

In conclusion, *LPAR3*, *ADORA1*, *GPR17*, and *OPRM1* may serve as therapeutic targets of ischemic stroke. Regulatory axis, such as SND1-IT1/NAPA-AS1/LINC01001-miR-24-3p-*LPAR3*/*ADORA1* and LUCAT1/ASAP1-IT2-miR-93-3p*-GPR17* may play important roles in the progression of ischemic stroke.

## Data Availability Statement

The original contributions presented in the study are included in the article/Supplementary Material, further inquiries can be directed to the corresponding author/s.

## Author Contributions

YS conceived and designed the research and drafted the manuscript. JW acquired the data. KM analyzed and interpreted the data. BH performed statistical analysis. YD revised the manuscript for important intellectual content. All authors read and approved the final manuscript.

## Conflict of Interest

The authors declare that the research was conducted in the absence of any commercial or financial relationships that could be construed as a potential conflict of interest.

## Publisher’s Note

All claims expressed in this article are solely those of the authors and do not necessarily represent those of their affiliated organizations, or those of the publisher, the editors and the reviewers. Any product that may be evaluated in this article, or claim that may be made by its manufacturer, is not guaranteed or endorsed by the publisher.

## Footnotes

1
http://www.ncbi.nlm.nih.gov/geo/

2
https://www.gencodegenes.org/human/

3
https://bioconductor.org/packages/limma/

4
https://string-db.org/

5
https://cytoscape.org/

6
http://apps.cytoscape.org/apps/mcode

7
https://bioconductor.org/packages/clusterProfiler/

8
http://www.dgidb.org/

9
https://www.grnpedia.org/trrust/
