# Supplementary material for: Elucidating the Molecular Mechanism of Ischemic Stroke Using Integrated Analysis of miRNA, mRNA, and lncRNA Expression Profiles
Source: Front Integr Neurosci. 2021 Aug 16;15:638114. doi: 10.3389/fnint.2021.638114 (PMC8415716; doi:10.3389/fnint.2021.638114)
Supplement: Supplementary file 1 [file Table_1.DOCX]

Supplementary table 1. Primer information.

| RNA | Primer sequence (5’-3’) |
| --- | --- |
| GPR17 | Forward sequence AGGATGTCCAAACGGAGTTGG |
|  | Reverse sequence GGCCATTCATGCTTTGGCTG |
| ADORA1 | Forward sequence CCTGGCCCTGCTGGC |
|  | Reverse sequence CACCATCTTGTACCGGAGAGG |
| OPRM1 | Forward sequence CATCACGATCATGGCCCTCTAC |
|  | Reverse sequence TCTGCCAGAGCAAGGTTGAAA |
| LPAR3 | Forward sequence CCGCATACAAGTGGGTCCAT |
|  | Reverse sequence GTCCAGCATACCACAAACGC |
| GAPDH | Forward sequence AATATGATTCCACCCATGGCAAAT |
|  | Reverse sequence CCCCACTTGATTTTGGAGGGA |
| SNHG5 | Forward sequence CGTTCTGAGTGTGGACGAGT |
|  | Reverse sequence TCCAAGACAATCTGGCCTCT |
| NAPA-AS1 | Forward sequence TTGGCCGCAATCGTGAAT |
|  | Reverse sequence GAACTTGAAGGGTCATAGGTCAG |
| SND1-IT1 | Forward sequence TCTCCAGAGGCTAGTGTTCC |
|  | Reverse sequence GTTCCAAGGAGGCCAAATCTC |
| U6 | Forward sequence CTCGCTTCGGCAGCACA |
|  | Reverse sequence AACGCTTCACGAATTTGCGT |
| miR-3135b | Forward sequence GGCTGGAGCGAGTGCA |
|  | RT GTCGTATCCAGTGCAGGGTCCGAGGTATTCGCACTGGATACGACCACCAC |
| miR-24-3p | Forward sequence GGTGGCTCAGTTCAGCAG |
|  | RT GTCGTATCCAGTGCAGGGTCCGAGGTATTCGCACTGGATACGACCTGTTC |
| miR-93-3p | Forward sequence GACACTGCTGAGCTAGCAC |
|  | RT GTCGTATCCAGTGCAGGGTCCGAGGTATTCGCACTGGATACGACCGGGAA |
| Universal reverse primer | GTGCAGGGTCCGAGGT |
